# Exponential Combination of *a* and *e/g* Intracellular Peptide Libraries Identifies a Selective ATF3 Inhibitor

**DOI:** 10.1021/acschembio.3c00779

**Published:** 2024-02-27

**Authors:** Miao Yu, T.M. Simon Tang, Lila Ghamsari, Graham Yuen, Claudio Scuoppo, Jim A. Rotolo, Barry J. Kappel, Jody M. Mason

**Affiliations:** † Department of Life Sciences, University of Bath, Claverton Down, Bath BA2 7AY, United Kingdom; ‡ Sapience Therapeutics, Inc. 500 Mamaroneck Ave. Suite 320, Tarrytown, New York 10591, United States

## Abstract

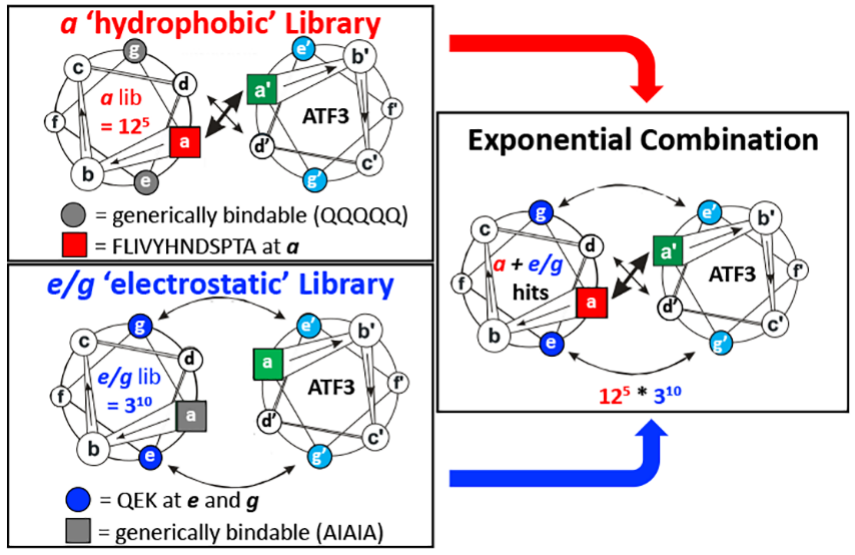

Activating transcription factor 3 (ATF3) is an activation transcription factor/cyclic adenosine monophosphate (cAMP) responsive element-binding (CREB) protein family member. It is recognized as an important regulator of cancer progression by repressing expression of key inflammatory factors such as interferon-γ and chemokine (C–C motif) ligand 4 (CCL4). Here, we describe a novel library screening approach that probes individual leucine zipper components before combining them to search exponentially larger sequence spaces not normally accessible to intracellular screening. To do so, we employ two individual semirational library design approaches and screen using a protein-fragment complementation assay (PCA). First, a 248,832-member library explored 12 amino acid positions at all five *a* positions to identify those that provided improved binding, with all *e/g* positions fixed as Q, placing selection pressure onto the library options provided. Next, a 59,049-member library probed all ten *e/g* positions with 3 options. Similarly, during *e/g* library screening, *a* positions were locked into a generically bindable sequence pattern (AIAIA), weakly favoring leucine zipper formation, while placing selection pressure onto *e/g* options provided. The combined *a/e/g* library represents ∼14.7 billion members, with the resulting peptide, ATF3W_aeg, binding ATF3 with high affinity (*T*_m_ = 60 °C; *K*_d_ = 151 nM) while strongly disfavoring homodimerization. Moreover, ATF3W_aeg is notably improved over component PCA hits, with target specificity found to be driven predominantly by electrostatic interactions. The combined *a/e/g* exponential library screening approach provides a robust, accelerated platform for exploring larger peptide libraries, toward derivation of potent yet selective antagonists that avoid homoassociation to provide new insight into rational peptide design.

## Introduction

**Figure 1:**
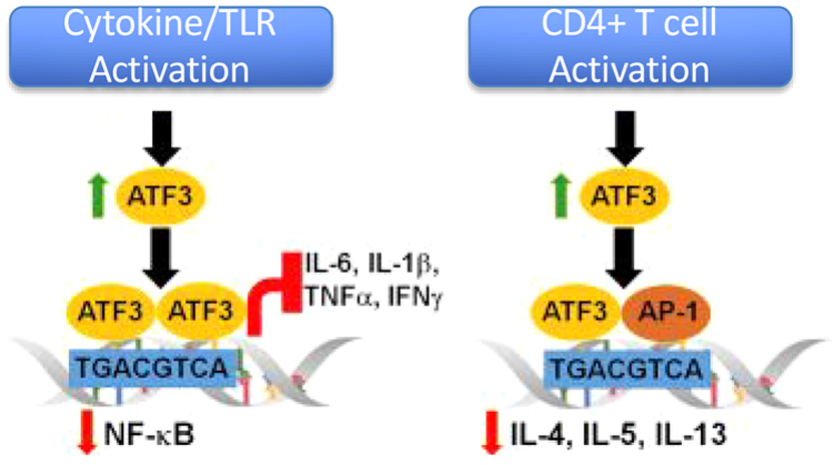
Overview of ATF3 activity in cancer. ATF3 plays an important role in host defense by regulating immune responses and cancer progression. In inactive immune cells, that include macrophages, natural killer cells, and CD4+ cells, it is expressed at low levels. Once these cells are activated by various signaling pathways including by TLRs (Toll-Like Receptors), cytokines, and antigen presentation, they become rapidly induced. ATF3 then binds to target promoters to regulate transcription. It appears to directly antagonize NF-kB and AP-1 driven promoters, resulting in reduced expression of certain cytokines. Controversially, ATF3 is also proposed to play a role as both a tumor suppressor and an oncogene. Figure adapted with permission from ref (7). Copyright Keai publishing 2017.

Activating transcription factor 3 (ATF3), a member of the mammalian ATF/cyclic adenosine monophosphate (cAMP) responsive element-binding (CREB) protein family, is a stress-induced transcription factor implicated in the modulation of immunity and oncogenesis in various cancers, including prostate, breast, colon, lung, and liver cancers.^1^ ATF3 homodimerizes or forms heterodimers with various partners via interactions between basic leucine zipper (bZIP) domains that bind the consensus cAMP response element (5-GTGACGT[AC][AG]-3) with varying affinities. Generally, homodimeric ATF3 serves to repress transcription of target genes that are involved in immune modulation, including interferon γ, chemokine (C–C motif) ligand 4 (CCL4), and E-selectin, thereby impairing macrophage migration and the recruitment of immune cells. ATF3 similarly plays an important role in repressing IL-6, IL-12, and other cytokine genes downstream of Toll-like receptor 4 (TLR4), by directly antagonizing NF-κB and AP-1 driven promoters and providing negative feedback to contain excessive inflammatory responses (Figure 1).

Expression of ATF3 is induced by a range of intra- or extracellular signals, causing it to form dimers which either activate or repress gene expression depending on the condition of the cell and promoter by transmitting signals from different receptors.^2^ ATF3 is a direct target of the Wnt/β-catenin pathway, where tumor-intrinsic Wnt/β-catenin activation induces ATF3 expression and inhibits the transcription of the pro-inflammatory chemokine macrophage inflammatory protein (MIP)-1β (also referred to as CCL4).^3^ The consequence of this signaling cascade is reduced tumor infiltration and activation of CD103+ dendritic cells, resulting in resistance to checkpoint blockade due to reduced CD8+ T cell priming and infiltration.^4^ In certain cancers, ATF3 has been shown to display oncogenic behavior via induction of metastatic mediators FN-1, TWIST-1, and Slug or suppression of GADD153, a known pro-apoptotic gene.^5^ Alternatively, ATF3 has been shown to display tumor suppressing activities by repressing transcription of cell cycle genes (such as cyclin D1 and ID1) and cell survival genes (such as IRS2).^6^ Selective ATF3 antagonist peptides therefore represent powerful potential tools to elucidate the role of ATF3 in cancer progression and a novel approach to enhance antitumor immunity in the context of many cancer types. Targeting bZIP transcriptional regulators such as ATF3, however, have presented a significant challenge. This class of protein–protein interaction (PPI) lacks the requisite hot spots for high-affinity or selective binding by small molecules. Additionally, ATF3 predominantly locates and functions in the nucleus, rendering targeting via larger biologics such as antibodies technically impractical. To overcome these obstacles, an alternative approach is to selectively target ATF3 using designed peptide inhibitors and abrogate ATF3 oncogenic function directly.

ATF3 dimerization via the leucine zipper motif is essential for DNA binding and represents a novel approach to antagonize a previously undruggable target. We focus on the selective targeting of the ATF3 leucine zipper dimerization domain using intracellular peptide library screening. The leucine zipper provides a direct link between the primary sequence and the highly specific nature of the quaternary structure vital to ATF3 dimerization and activity. The periodic pattern of hydrophobic and electrostatic interactions within heptad repeats observed in coiled coil dimers is well-defined and provides confidence in a library design approach. To derive a selective peptide that binds and disrupts the ATF3 homodimer without itself homodimerizing, we utilized the combination of two distinct semirational library design approaches that focus on the core *a* and flanking *e/g* components, respectively. The approach is highly appealing in that two smaller libraries accessible to intracellular screening platforms can be individually probed and then combined to sample sequence spaces that are exponentially larger than either component. The approach has been employed to enable the identification of a library member with high affinity and selectivity for the oncogenic immune modulating transcription factor ATF3.

## Results and Discussion

### Library *a* and *e/g* Design and Protein-Fragment Complementation Assay (PCA) Strategy

**Figure 2:**
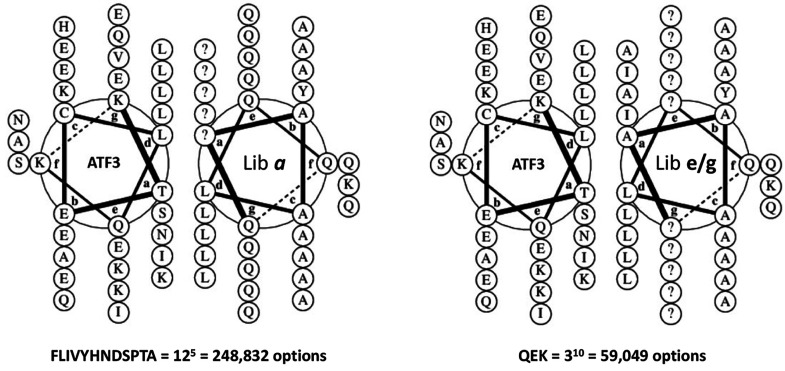
ATF3 library *a* and library *e/g* design and screening presented in the diagrams of helical wheels. Options at each position within Lib *a* were FLIVYHNDSPTA. Options at each position within Lib *eg* were QEK. Helical wheel diagrams were generated using DrawCoil 1.0, https://grigoryanlab.org/drawcoil/.^11^

**Figure 3:**
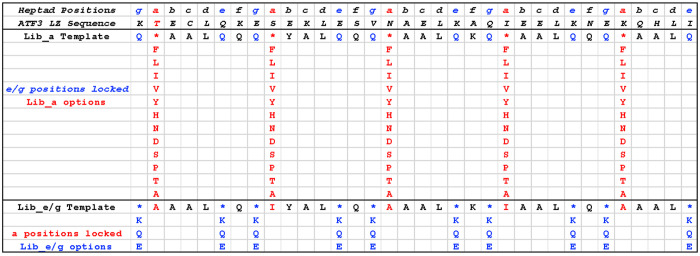
ATF3 library *a* and *e/g* designs. During library design for PCA screening, peptide options were semirandomized. Lib_a: all five *a* positions were semirandomized to provide 12 options (FLIVYHNDSPTA^5^ = 248,832 options) while all 10 *e/g* positions were fixed as Q to provide a generic electrostatic option predicted to provide weak nonselective affinity. Lib_e/g: All 10 *e/g* positions were semirandomized to provide 3 options (QKE^10^ = 59,049 members) while core *a* positions were fixed as AIAIA to facilitate parallel dimeric coiled coil formation of low affinity to place a strong selection emphasis upon the electrostatic component. Shown are hydrophobic options at core interfacial positions (*a*) and charged/polar options which are present at flanking positions (*e/g*). In particular, the *g* and *e* positions have been semirandomized to provide attractive and repulsive options with corresponding positions on the target. Likewise, interfacial *a* positions were semirandomized to generate a range of options that included aliphatic hydrophobic side chains, as well as Asn, Ala, and aromatic options (Phe, Tyr, His). Positions *c* and *d* positions were fixed as A and L, respectively (position *b2* was fixed as Y for quantification purposes). The appeal of the approach is that the two libraries can be exponentially combined to produce a library size of ∼14.7 billion members that is inaccessible to intracellular screening platforms.

We describe two individual semirational library designs that allow for a pairwise exponentially large library to be probed when combined. This involves one library with 12 residue options at all five *a* positions (12^5^ = 248,832 members, importantly containing the most typical coiled coil forming residues L, I, V, A, N, in addition to options F, Y, H, D, S, P, T) and a second library with Q/E/K options (polar/negative/positive) typically found across all ten *e/g* residues of the LZ (3^10^ = 59,049) to provide a combined library of almost 1.5e^10^ sequences. Both libraries were individually screened against ATF3 by PCA and hit sequences were combined to give an effective antagonist (Figures 2 and 3). Overall, the approach gives high confidence in both the screening elements and the subsequent combining of individual library elements.

Library *a* focused entirely on the *a* position at the core of the coiled coil to identify residues providing optimal hydrophobic packing at the coiled coil dimer interface and thus LZ stability. To direct selection pressure onto the core *a* residues, the role of electrostatics interactions in LZ dimerization was de-emphasized by fixing all *e/g* positions as Q, an amino acid that has been shown to be generically favorable at these positions, being neither favored nor disfavored for many opposing residues (e.g., K, Q, R, E) typically located at corresponding positions on the target helix.^8^

The second *e/g* library was next probed to identify the most appropriate electrostatic interactions to engage the ATF3 target. In this library, the role of the core *a* position similarly was de-emphasized to provide only a weak overall contribution to dimer formation. To achieve this, hydrophobic bulk was removed from the core to ensure formation of a parallel dimer, without this element being a key driver (i.e., a1–a5 = AIAIA).^9,10^ Two Ile residues provided sufficient favored hydrophobicity to promote LZ formation but were insufficient for the core alone to dictate high affinity binding. The placement of these residues at position *a* (AIAIA) pushes the selection pressure onto the *e/g* residues during the screening process. Recombination of the two hits from distinct in-cell PCA screens yielded a selective antagonist from the exponential sampling of the two libraries. During library building and selection, library accuracy and residue variations were verified by DNA sequencing (Figure S1).

### PCA Screening and Selection

**Figure 4:**
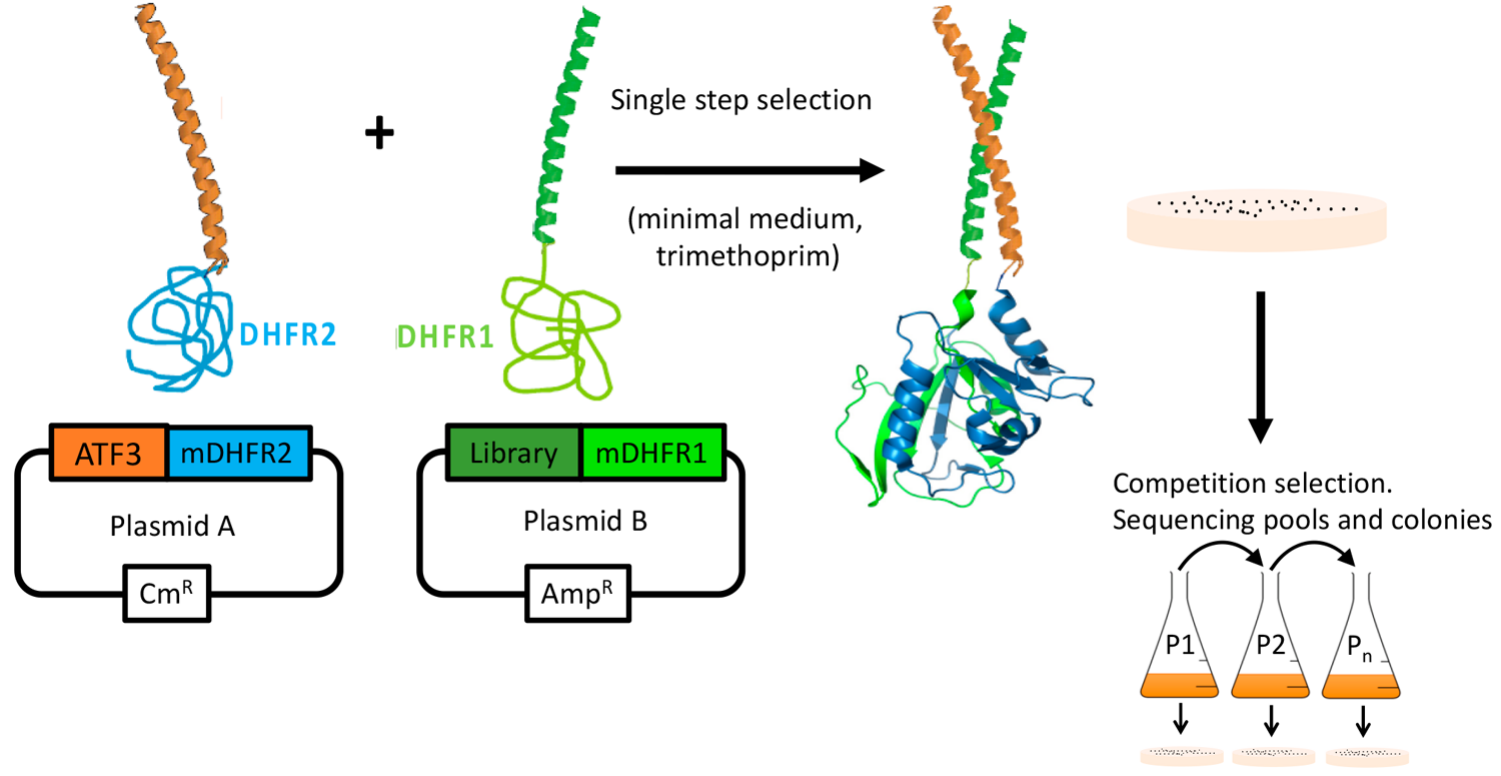
Protein-fragment complementation assay. Both library *a* and *e/g* selections were carried out in bacteria. During PCA, members that bind to the ATF3 leucine zipper result in the recombination of the murine dihydrofolate reductase (mDHFR) enzyme, leading to the generation of colonies under M9 selective conditions (with bacterial DHFR selectively inhibited using the antibiotic trimethoprim). Those peptides displaying the highest affinity for ATF3 conferred the fastest cell growth rates. Subsequent competition selection passages were then undertaken in liquid medium to enrich potential PCA winners with the highest efficacy. PCA is additionally performed in the cytoplasm of *E. coli*, meaning that nonspecific, toxic, unstable, aggregation-prone (insoluble), and protease-susceptible members are removed.

During PCA, half of the enzyme murine dihydrofolate reductase (mDHFR) was genetically fused to the ATF3 target, with the second part fused to the protein library. During PCA selection, only those library members capable of binding to the ATF3 target, within the complex intracellular environment, resulted in a recombination of the two mDHFR halves, rendering the enzyme active under selective conditions leading to bacterial colony formation (Figure 4). For both libraries, single step PCA selection was carried out using M9 agar plates under selective conditions and was followed by competition selection in liquid M9 medium to enrich for the most effective sequences. This process resulted in one clean sequence after seven serial passages for Library *a* selection: QLAALQQQAYALQQQNAALQKQVAALQQQIAALQ, ATF3W_a; one clean sequencing for Library *e/g* selection: EAAALEQKIYALKQEAAALEKEIAALEQKAAALK, ATF3W_eg. The selected sequences from the two library screens were combined. The resultant combined peptide (ELAALEQKAYALKQENAALEKEVAALEQKIAALK, ATF3W_aeg) represents the most effective ATF3 binding sequence from 14.7 billion peptides (12^5^ × 3^10^) via an exponential sampling of the two libraries. DNA sequencing results from PCA library pools and individual colonies are presented in Figure S2.

**Figure 5:**
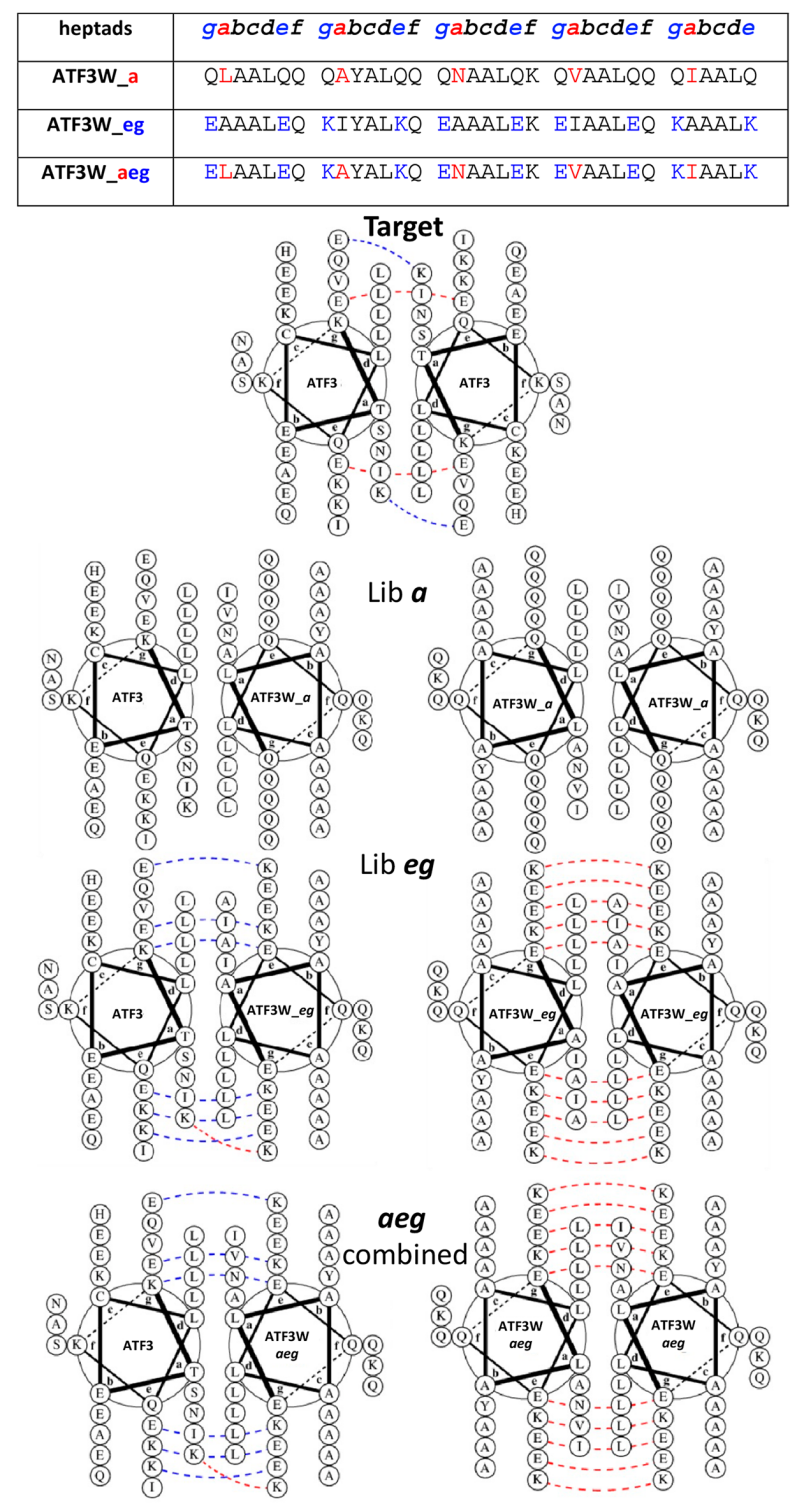
Helical wheel representations of potential interactions with the PCA selected ATF3W_a and ATF3W_eg sequences as well as combined sequence ATF3W_aeg. ATF3-ATF3W_aeg heterodimeric and ATF3W_aeg homodimeric helical wheel diagrams show hydrophobic residues at core positions (*a/d*) as well as charged residues present at the surrounding positions (*e/g*). The *d* positions were held as Leu in order to maintain the leucine zipper structure. The ATF3-ATF3W_aeg interaction contains favorable electrostatic (blue dashed line) and core interactions to drive formation of the coiled coil. In contrast, the ATF3W_aeg dimer displays unfavorable electrostatic interactions (red dashed line) and van der Waals interactions with the core, disfavoring its formation. Helical wheel diagrams were generated using DrawCoil 1.0, https://grigoryanlab.org/drawcoil/.^11^

Helical wheel projections of the selected peptides were inspected to identify potential homo- and heterodimer interactions toward the target ATF3 (Figure 5). These diagrams show the hydrophobic interface and core positions (*a/d*) as well as electrostatic or polar residues present at the surrounding positions (*e/g*). Leu residues at core *d* positions were left unchanged to maintain both the parallel nature and dimeric oligomeric state of the leucine zipper structure. The ATF3-ATF3W_aeg heterodimer contains six favorable E–K electrostatic interactions between *e* and *g* positions. In contrast, the ATF3W_aeg homodimer contains ten unfavorable electrostatic interactions (6 E–E and 4 K–K). Similarly, the ATF3 target homodimer contains two electrostatic E–E repulsions. Therefore, the electrostatic component of ATF3W_aeg provides a much greater scope for on-target stabilization, while readily overcoming potential target dimers and avoiding homodimerization, freeing the molecule for target engagement and enhancing interaction specificity. Core residues within ATF3 deviate from designed “peptide Velcro” leucine zippers, are less favorable for hydrophobic packing, and are more difficult targets in terms of intuitive library design options. In particular, the ATF3 core *a* positions consist of T/S/K at *a1*, *a2*, and *a5* respectively. Only N and I at positions *a3* and *a4* are characteristic of a typical leucine zipper. A key strength of the combined Library *a* and Library *e/g* approach is that a broader range of amino acids can be explored for the requisite directed evolution to occur, particularly at the positions where residues capable of forming desired on-target interactions are harder to predict. Within the 12 options, canonical L/I/V/A/N options were provided, as well as aromatic alternatives F/Y/H (and P via unavoidable coding), and polar options N/D/S/T. The broad options provided mean that library *a* can sample a greater peptide space to engage the unusual ATF3 core arrangement by potentially selecting residues seldom observed at the core of coiled coils. Interesting, mostly canonical core residues were selected, with the small side chain of Ala selected at positions *a2* to accommodate S/T of ATF3. Alanine selection is also expected to destabilize the potential for ATF3W_aeg homodimer formation. In addition, Ile was selected at position *a5* and is in proximity of an *a5* K in ATF3. Reassuringly, from the 12 options provided, N was selected at *a3* where it is predicted to guide interaction specificity via formation of an N–N hydrogen bond with a corresponding N at *a3* witin ATF3.^12^

### Circular Dichroism Studies

**Figure 6:**
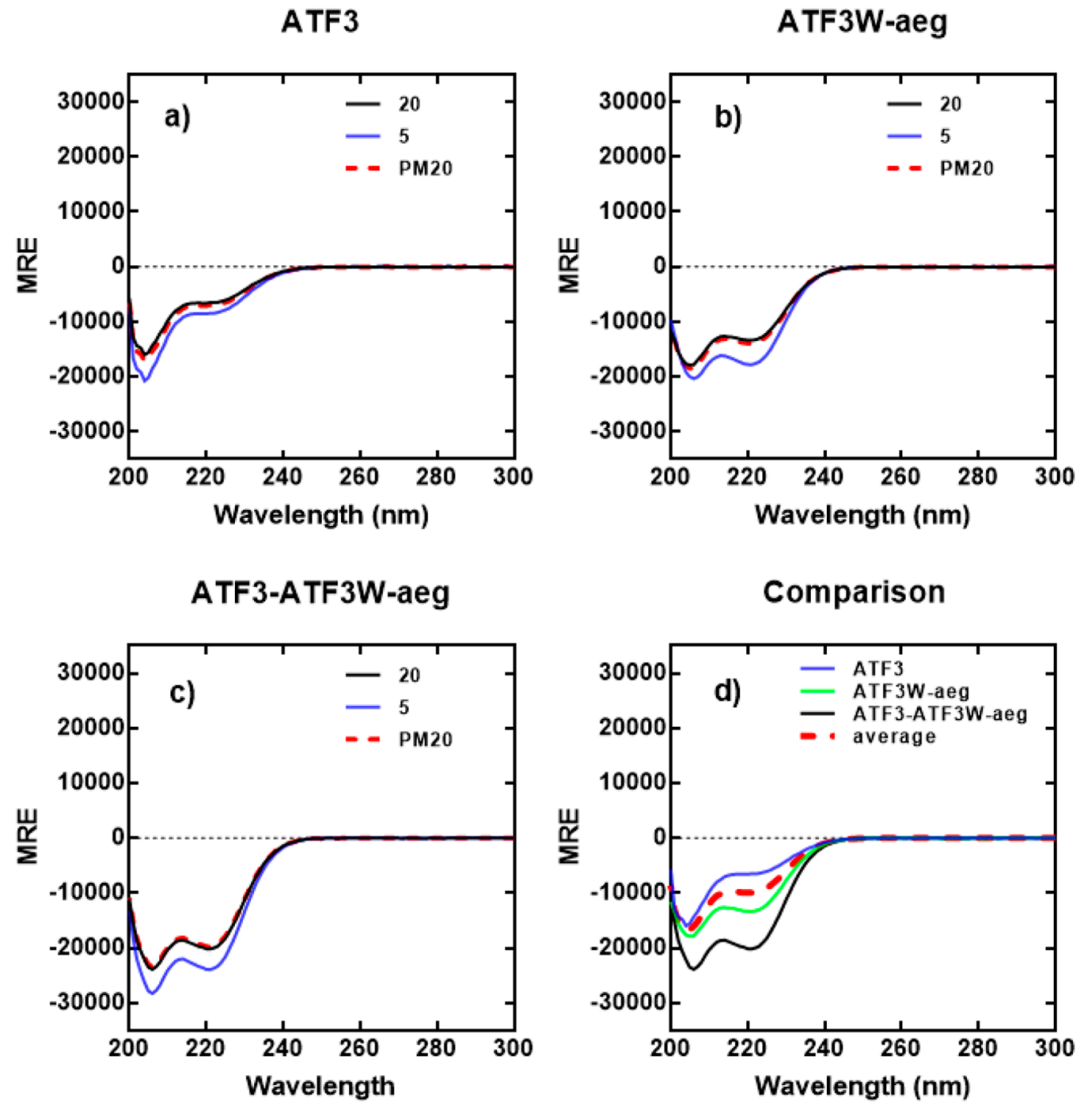
CD spectra data indicates a ATF3-ATF3W_aeg interaction. CD spectra are shown for (a) ATF3, (b) ATF3W_aeg, and (c) ATF3-ATF3W_aeg, with all samples premixed at 1:1 stoichiometry. Spectra were next measured at 20 and 5 °C and again post-thermal denaturation (PM 20 °C to establish reversible that unfolding is fully reversible) at a total peptide concentration of 150 μM and presented as mean residue ellipticity (MRE). (d) CD spectra are shown at 20 °C for ATF3 and ATF3W_aeg alone and mixed, the latter demonstrating a significant gain in measured signal (black) over the average of the two component signals (red hash). All spectra are indicative of helical structures. All experiments were performed in 10 mM potassium phosphate and 100 mM potassium fluoride (pH 7.0). CD spectra for interactions with component ATF3W_a and ATF3W_eg peptides are shown in the Supporting Information.

Following helical wheel inspection of PCA hits, we next sought to demonstrate both ATF3 target-binding and selectivity, relative to potential ATF3 and winner peptide homodimers. ATF3, ATF3W_a, and ATF3W_eg and combined hit sequence ATF3W_aeg were synthesized using solid phase peptide synthesis, purified by RP-HPLC, and verified for correct mass using ESI-MS (Figure S3), after which circular dichroism (CD) was used to characterize both ATF3 and antagonist peptides for helicity and interactions as homodimers, as well as heterodimers with the ATF3 target. Global secondary analysis of both homodimeric and heterodimeric systems was carried out at a total peptide concentration of 150 μM to keep equimolar concentrations of each component helix. The 222/208 ratio was used to provide evidence on whether the helices were likely to be monomeric or adopt quaternary structure.^13^ The CD spectra confirmed that all samples displayed varying degrees of α-helical signal, with both ATF3 (Figure 6a) and ATF3W_aeg (Figure 6b) in isolation existing as weakly populated helical structures (∼20% and ∼38% fH, respectively), with 222/208 ratios both significantly lower than 1 at 20 °C (0.49 and 0.76, respectively), further suggesting the presence of a monomeric helix. In contrast, the ATF3-ATF3W_aeg complex (Figure 6c) displayed a significant increase in α-helical signature (∼68% fH), more than 3 times that of the target ATF3, and an 222/208 ratio of 0.94, providing further evidence toward a significant increase in ATF3-ATF3W_aeg stability. Furthermore, incubation of ATF3 with ATF3W_aeg elicited a significant conformational change in the sample relative to the average of the component peptides (Figure 6d black line vs red hash), providing compelling evidence for an interaction.^14^ In addition, the component PCA hits were examined using CD. The data for ATF3 with ATF3W_a and ATF3W_eg, respectively, are shown in Figure S4. These data show in both cases that the heterodimers are more stable than the average of the component helices but less pronounced than that of the combined ATF3-ATF3W_aeg heterodimer.

### Thermal Denaturation Profiles

**Figure 7:**
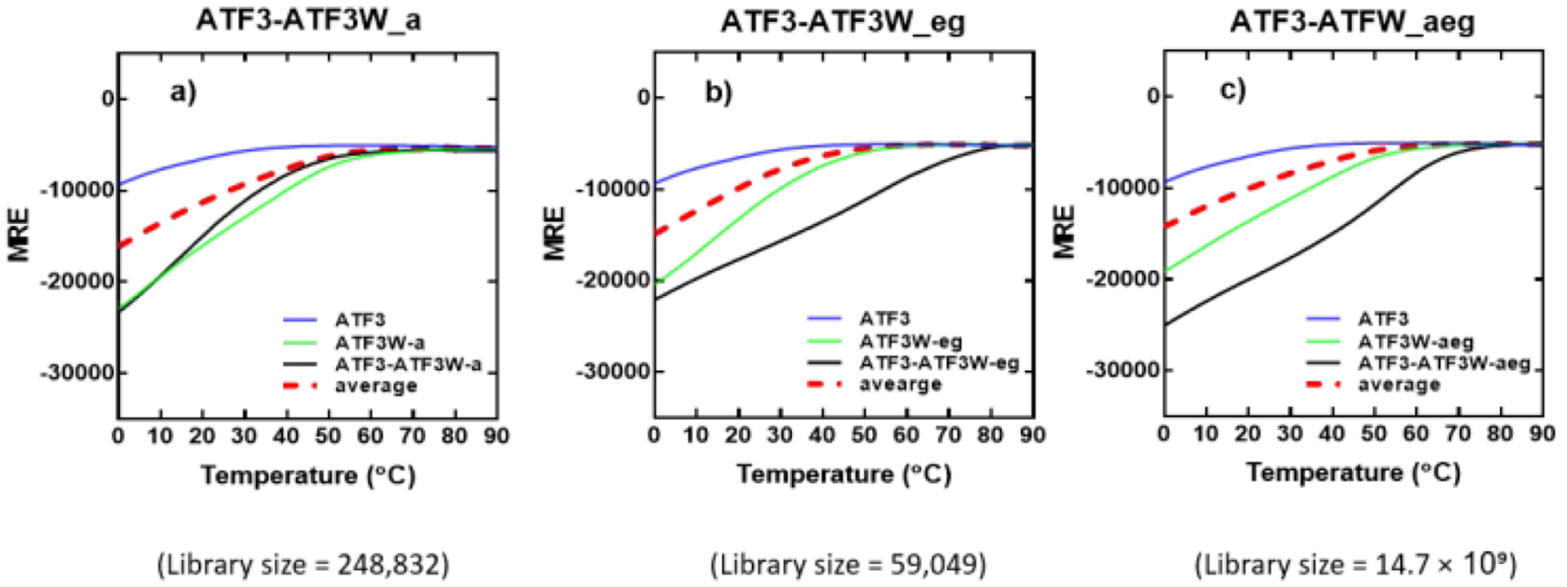
CD thermal denaturation profiles demonstrate that ATF3W_aeg interacts with ATF3. Shown are thermal stabilities of peptide pairs measured via temperature dependence of the CD signal at 222 nm. The thermal denaturation profiles for (a) ATF3-ATF3W_a, (b) ATF3-ATF3W_eg, and (c) ATF3-ATF3W_aeg show a substantial increase in the transition midpoint (*T*_m_ = 59 °C) relative to component peptides (black vs red hash). Experiments were performed in 10 mM potassium phosphate and 100 mM potassium fluoride (pH 7.0). All spectra were recorded at 1 °C increments at a total peptide concentration of 150 μM and fitted to a two-state denaturation model.

Following the observed significant increase in global secondary structure content for the ATF3-ATF3W_aeg complex relative to component peptides (Figure 6), we next analyzed the stability of the complex by undertaking thermal denaturation experiments (Figure 7). In agreement with the spectra, the desired complexes exhibit increased thermal stability. Only the upper baseline characteristic of the denaturation profile was observed when ATF3 was examined in isolation (Figure 7, blue), suggesting that ATF3 was unable to self-assemble into a complex. In contrast, when ATF3 was incubated with ATF3W_a (Figure 7a, green), ATF3W_eg (Figure 7b, green), or the combined molecule ATF3W_aeg (Figure 7c, green), the intensity of the helical signal increased significantly, leading to increased transition midpoints of 26, 52, and 60 °C, respectively (Figure 7, black). The inability of ATF3W_aeg to form a stable homodimer in isolation is a major advantage inherited from the ATF3W_eg component peptide, since it provides electrostatic repulsion that disfavors the homodimeric complex as a potential off-target interaction, freeing the molecule to adopt a dimerization competent state with the target. Moreover, consistent with spectral data, the clear increase from the average profile of the component peptides relative to that of the measured is most pronounced in the combined construct (Figure 7, red hash vs black). This provides robust additional evidence for a preferential interaction between ATF3 and ATF3W_aeg that is improved over either the core *a* or electrostatic *e/g* constructs.

### Size-Exclusion Chromatography (SEC)

**Figure 8:**
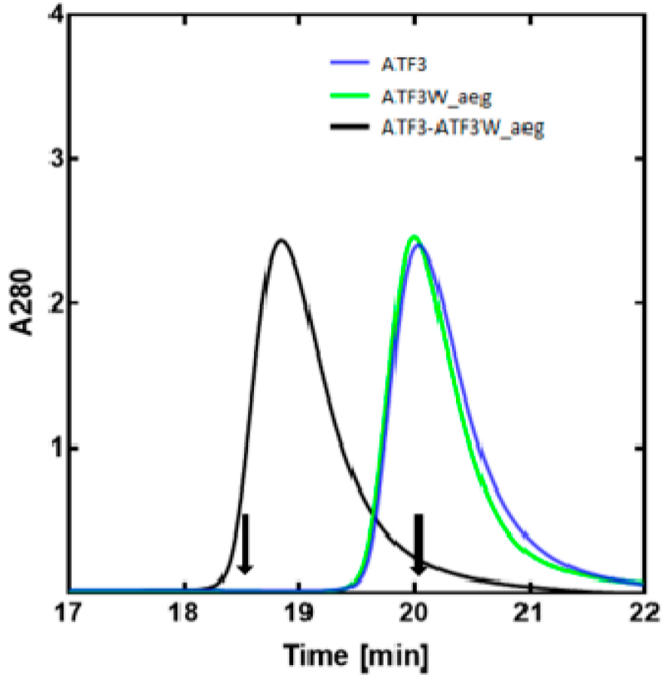
SEC profiles indicate that ATF3W_aeg binds ATF3. Shown are size exclusion chromatography profiles for interacting peptides. The peak at approx 19 min for the ATF3-ATF3W_aeg mixture (black trace) represents a dimeric sample while ATF3 (blue trace) and ATF3W_aeg (green trace) generate a peak at approx 20 min, indicating the presence of a monomer. These experiments, undertaken at a total peptide concentration of 20 μM, provide additional evidence for selectivity of the ATF3-ATF3W_aeg interaction. Arrows show controls from previous peptides with elution times for a 32 mer Fos monomeric peptide (20 min) and a 37 mer cJun–FosW heterodimer (18.5 min).^15^

To further demonstrate correct peptide pairing as well as oligomeric state, size-exclusion chromatography (SEC) was employed. During SEC experiments, monomeric ATF3 (Figure 8, blue line) and ATF3W_aeg (Figure 8, green line) eluted at approximately 20 min. These contrast with the dimeric profile observed for ATF3-ATF3W_aeg (Figure 8, black line), which occurred at approximately 19 min. For all samples, elution profiles were consistent with either a validated monomer or dimer elution pattern.^15^ SEC experiments therefore provide further evidence for correct pairing of the combined peptide by demonstrating that (i) ATF3W_aeg exists exclusively as a monomer in solution and (ii) an interaction between ATF3 and ATF3W_aeg was formed that was (iii) exclusively dimeric in nature.

### Isothermal Titration Calorimetry (ITC)

**Figure 9:**
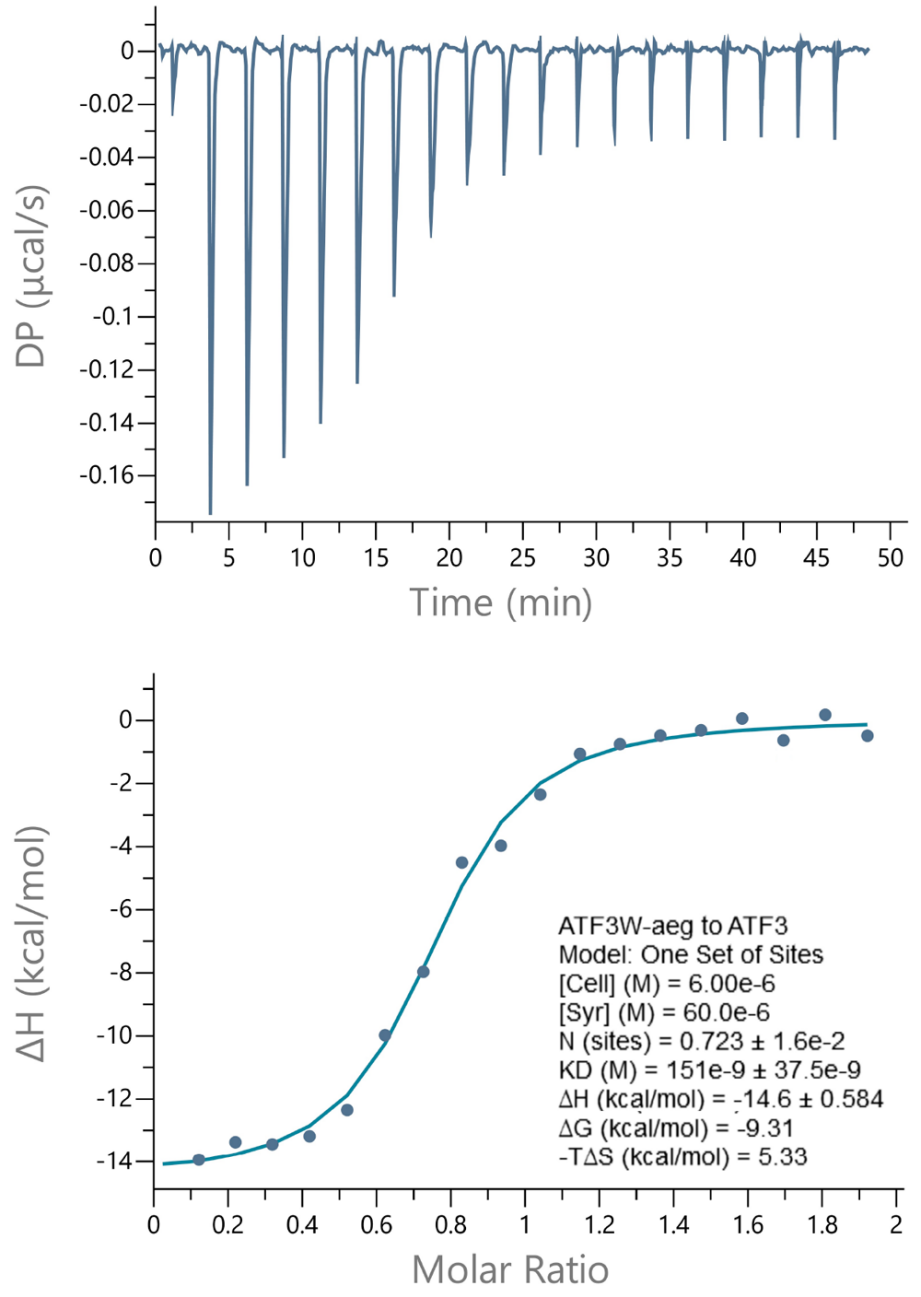
Isothermal titration calorimetry analysis of the ATF3-ATF3W_aeg interaction. In the bottom plot, the solid line represents the fit to the data based on the binding of a ligand to a macromolecule using the MicroCal (GE Healthcare) Origin software.^16^ See Materials and Methods for further details.

To provide additional insight into the origin of the binding affinity (*K*_D_) between ATF3 and ATF3W_aeg, isothermal titration calorimetry (ITC) experiments were performed. ITC enables the free energy of binding to be deconvoluted into entropic and enthalpic components (Figure 9), while also providing a stoichiometric measure of binding that further demonstrates the population of a dimer (*N* = 0.72). Thermodynamic parameters determined from ITC measurements on ATF3-ATF3W_aeg further confirmed the interaction; titrating a solution of 60 μM ATF3W_aeg into that of 5 μM ATF3 elicited the expected sigmoidal binding curve, with the fit deriving a *K*_D_ of 151 nM (Δ*G* = −9.31 kcal/mol). The free energy of binding was predominantly driven by a favorable enthalpic term (Δ*H* = −14.6 kcal/mol) and opposed by an unfavorable entropic component (*T*Δ*S*= −5.33 kcal/mol).

### THP-1 Polarization Assay and CCL-4 ELISA

**Figure 10:**
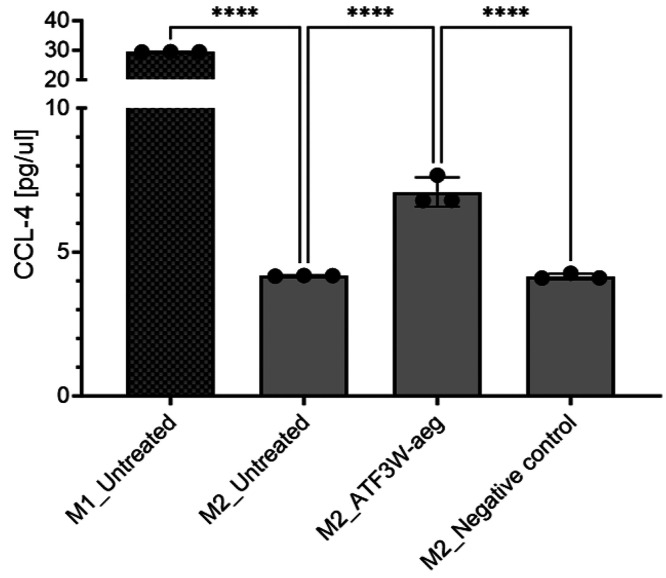
ATF3W-aeg peptide stimulates expression of pro-inflammatory cytokine in M2 macrophages. CCL4 protein levels were measured by ELISA from supernatants of M2 macrophage cultures treated with either 20 μM ATF3W-aeg or negative control peptide (****p < 0.0001). Shown also are CCL-4 protein levels in M1 (pro-inflammatory) macrophages.

ATF3 is a transcriptional repressor that regulates both the extent and duration of pro-inflammatory gene expression.^17^ Specifically, ATF3 has been implicated in the transcriptional repression of chemokines including chemokine (C–C motif) ligand 4 (CCL4),^18^ also known as macrophage inflammatory protein (MIP-1β), which promotes recruitment of additional immune cells to inflamed tissues. In the context of cancer, ATF3 activity counters antitumor immunity and promotes tumor growth. We therefore explored the effect of the ATF3W-aeg antagonist peptide on CCL4 expression in M2 anti-inflammatory macrophages differentiated from the human THP-1 monocytic cell line. M2 macrophages were polarized and then either treated with ATF3W-aeg peptide, a negative control peptide, or left untreated for 48 h. Treatment of M2 macrophages with ATF3W-aeg significantly increased CCL4 expression, indicating inhibition of ATF3 activity, while the control peptide had no effect (Figure 10). These findings demonstrate that the ATF3W-aeg antagonist peptide can modulate CCL4 expression levels in human macrophages.

## Conclusions

We describe an approach of employing two semirational libraries that separately screen the *a* and *e/g* positions of a component helix within a leucine zipper. By employing generic and weakly binding residues such as Q at *e/g* positions or AIAIA at *a* positions, the selection pressure was directed toward the scrambled positions in the *a* and *e/g* libraries, respectively. This also includes the possibility to direct the component libraries against other LZ targets. Consequently, the hydrophobic and electrostatic drivers of the desired binding to ATF3 were optimized individually. Selected peptide sequences from the two semirational libraries were next combined to generate a peptide representative of a hit derived from an exponentially greater sequence spaces (12^5^ × 3^10^ = ∼15 billion members) not normally accessible to intracellular PCA screening. This “split-and-pool” strategy allows us to tackle otherwise enormous sequence space which is inaccessible to the current intracellular screening capacity. A potential risk was the possibility that *e/g* and *a* changes might not have worked together when combined into one molecule, for example, with interactions between *d-e′* and *a-g′* residues,^19,20^ and that the combined changes might compromise binding. However, there is known to be minimal crosstalk between these paired interactions, and we have previously shown that *a-a′/d-d′* and *g-e′* interactions play the most significant role in pairing.^21^ Furthermore, since PCA is performed in the presence of the host proteome, nonspecific, toxic, unstable, aggregation-prone (insoluble), and protease-susceptible peptide library members are removed during screening. We find this separate screening approach to be a highly effective strategy for antagonist selection. Overall, the capability of the two screening elements to be recombined into one hybrid molecule with improved features over either component provides high confidence in their individual ability to probe distinct aspects of the peptide sequence for structure and binding to target. We therefore have high confidence in both screening elements and their ability to recombine synergistically into one molecule with improved features outperforming either component peptides. The ability to screen a more diverse set of amino acid options in library *a* is particularly powerful when the target (e.g., ATF3) contains residues at the core *a* position which deviate from the conventional coiled coil design paradigm. At the core, some ATF3 residues favor polar or hydrophobic interactions, with the *a* position consisting of N (*a3*) and I (*a4*). ATF3W_aeg takes advantage of this core arrangement with *a3* selected as N from the 12 options provided to capitalize on the oligomer-limiting locus of the *a3* N–N interaction.^12^ On the other hand, library *e/g* identified the most suitable electrostatic interactions to favor ATF3 binding while disfavoring off-target interactions. The ATF3-ATF3W_aeg heterodimer consists of six favorable electrostatic interactions between *e* and *g* residues, between oppositely charged E and K. In contrast, ATF3W_aeg homodimers are predicted to be destabilized via ten unfavorable K–K and E–E electrostatic repulsions, providing robust evidence that PCA encompasses this negative design element during selection. These provide a greater scope for stabilization of antagonist to target heterodimeric complexes and destabilize antagonist homodimers to free the molecule for effective target engagement providing enhanced interaction specificity.^22^ The *in vitro* analysis of the ATF3-ATF3W_aeg interaction further proves the stability and specificity of ATF3-ATF3W_aeg binding by CD and ITC as it has shown that the antagonist of ATF3W_aeg specifically interacted with the target ATF3 with a measured *K*_d_ of 151 nM and outcompeted the undesired self-binding of ATF3W_aeg. The free energy of binding is predominantly driven by a favorable enthalpic term (Δ*H* = −14.6 kcal/mol) which outweighs the unfavorable entropic component (*T*Δ*S*= −5.33 kcal/mol). This reinforces the design strategy and clearly shows that synergy can be gained by creation of a hybrid molecule from the *a* and *e/g* libraries. In the future, it may be possible to probe the role of these positions in concert with solvent exposed *b*, *c*, and *f* positions to further improve affinity via less direct mechanisms that maintain and improve α-helical propensity, helix solubility, intramolecular stability,^20^ and potentially cell permeability. In conclusion, the combined library *a* and library *e/g* multidisciplinary approaches represents a powerful approach to investigate and optimize individual components contributing to target binding, toward the derivation of new molecules and insights into rational drug design that can facilitate the search of selective inhibitors for ATF3 and other bZIPs in general.

## Materials and Methods

A 248,832-member peptide library *a* combined with a 59,049-member peptide library *e/g* was designed by introducing semirandomized residue options at positions corresponding to key interfacial positions within each heptad repeat of a coiled-coil motif (*gabcdef*). Each *g* or *e* position within the coiled coil, which is critical in forming electrostatic contacts within a coiled-coil sequence, was semirandomized to generate Q/E/K options, with a view to generate both potential attractive and repulsive options with the corresponding positions of the target (Figure 3) while the core was specifically designed as AIAIA to remove the core hydrophobicity. Similarly, all *a* positions corresponding to the core region within a coiled-coil sequence (*a1*, *a2*, *a3*, *a4*, *a5*) were semirandomized to generate F/L/I/V/Y/H/N/D/S/P/T/A options while all *e/g* positions were fixed to generic bindable Q. All *c* and *d* positions were fixed as A and L, respectively, to impart helicity and further core hydrophobicity that is characteristic of the parallel dimeric coiled-coil motif.^23,24^

### PCA and Expression Vector Cloning

PCA has been extensively used to derive PPI antagonists of activator protein.^23^ The ATF3 gene was synthesized by overlap extension PCR: ATF3 Forward: 5′-ATA ATA GCT AGC AAA ACC GAA TGC CTG CAG AAA GAA AGC GAA AAA CTG GAA AGC GTG AAC GCG GAA CTG AAA GCG CAG-3′; ATF3 Reverse: 5′-ATA ATA CGG CGC GCC AAT CAG ATG CTG TTT TTC GTT TTT CAG TTC TTC AAT CTG CGC TTT CAG TTC CGC-3′.

### PCA Library Construction

Primers to encode the desired library were generated using overlap-extension PCR.^23^ Primers used were ATF3-Lib*a*-Forward: 5′-ATT GCT AGC CAA NHC GCG GCA CTG CAG CAG CAA NHC TAT GCG CTG CAG CAG CAA NHC GCG GCC CTG CAG AAA CAG-3′; ATF3-Lib*a*-Reverse: 5′-AA AGG CGC GCC CTG CAG TGC CGC GDN TTG CTG CTG CAG TGC CGC GDN CTG TTT CTG CAG GGC CGC-3′; ATF3-Lib*e/g*-Forward: 5′-ATT GCT AGC VAG GCG GCG GCA CTG VAG CAG VAG ATC TAT GCG CTG VAG CAG VAG GCG GCG GCC CTG-3′; ATF3-Lib*e/g*-Reverse: 5′-AA AGG CGC GCC CTB CAG TGC CGC CGC CTB CTG CTB CAG TGC CGC AAT CTB TTT CTB CAG GGC CGC CGC C-3′.

### Single-Step Selection PCA

*Escherichia coli* XL-1 cells were used for construction and cloning of libraries as described previously.^21,23^

### Competition Selection PCA

To increase selection stringency, growth competition experiments were undertaken as described previously.^23^ Seven rounds of competition selection were performed before the pool was found to contain one clean sequence. Library *a* selection: QLAALQQQAYALQQQNAALQKQVAALQQQIAALQ; Library *e/g* selection: EAAALEQKIYALKQEAAALEKEIAALEQKAAALK. The final selected sequence combined two library selections at *a*, *e*, and *g* positions as ELAALEQKAYALKQENAALEKEVAALEQKIAALK and was named ATF3W_aeg.

### Peptide Synthesis

Peptide synthesis was undertaken as described previously.^23^ Following HPLC purification, collected fractions were examined by electrospray MS (Figure S3), with those containing pure product pooled and lyophilized. Post RP-HPLC analysis indicated a purity of >95%.

### Circular Dichroism

CD was carried out using an Applied Photophysics Chirascan CD apparatus (Leatherhead, UK) using a 200 μL sample in a CD cell with a 1 mm path length as described previously.^23^

### Thermal Denaturation Experiments

Thermal denaturation experiments were performed at 150 μM total protein concentration in 10 mM potassium phosphate and 100 mM potassium fluoride, pH 7, using an Applied Photophysics Chirascan CD instrument (Leatherhead, UK) as previously described.^23^

### Isothermal Titration Calorimetry (ITC)

ITC measurements were made using a Microcal PEAQ-ITC instrument with data collected and processed using the Origin 7.0 software package.^14,23^ All measurements were carried out at least twice. Briefly, all peptides were studied at 20 °C in 10 mM potassium phosphate and 100 mM potassium fluoride at pH 7.0. 40 μL of ATF3W_aeg was loaded into the syringe at 60 μM peptide concentration. 350 μL of ATF3 was loaded into the cell at 6 μM. The experiment was undertaken by injecting 2 μL of ATF3W_aeg 19 times into the calorimetric cell. Following ITC measurements, the data were fit to a one-site model.

### Size-Exclusion Chromatography

Size-exclusion experiments were performed at RT using a Superdex Peptide 10/300 GL column (GE Healthcare Life Sciences) as described previously.^23^

### THP-1 Polarization Assay and CCL-4 ELISA

THP-1 cells were seeded in 12-well format at a concentration of 5 × 10^5^ cells/well and treated with 7.5 ng/mL Phorbol 12-Myristate 13-Acetate (PMA, Sigma-Aldrich) in RPMI media supplemented with 10% FBS and 55 μM β-mercaptoethanol (BME). After 24 h, PMA-containing media were removed and replaced with fresh media. After three additional days, cells were polarized toward the M1 program by adding 250 ng/mL LPS (*E. coli* O111:B4, Sigma-Aldrich) and 20 ng/mL IFN-γ (R&D Systems) or the M2 program by adding 20 ng/mL IL-4 (R&D Systems). At the same time, 20 μM ATF3W-aeg with an NLS-TAT cell penetrating sequence and nuclear localization sequence appendage (Ac-ELAALEQKAYALKQENAALEKEVAALEQKIAALKPKKKRKVYGRKKRRQRRR-NH2) peptide or negative control (Fra1W-NLS TAT: Ac-KAAALKQKAYALKQQIAALKKQVAALKQKIAALKPKKKRKVYGRKKRRQRRR-NH2)^23^ were added. After 48 h, supernatants were collected and clarified by centrifugation, and the CCL-4 concentration was assessed by ELISA (MIP-1b Human Instant ELISA Kit, ThermoFisher Scientific) according to the manufacturer’s protocol.

## Supporting Information Available

Additional experimental details, materials, and methods, including library designs, DNA sequencing, and mass spectrometry data (PDF)

## References

[ref1] Ku H. C.; Cheng C. F. Master Regulator Activating Transcription Factor 3 (ATF3) in Metabolic Homeostasis and Cancer. Front Endocrinol (Lausanne) 2020, 11, 556 10.3389/fendo.2020.00556. 32922364 PMC7457002

[ref2] Rohini M.; Haritha Menon A.; Selvamurugan N. Role of activating transcription factor 3 and its interacting proteins under physiological and pathological conditions. Int. J. Biol. Macromol. 2018, 120 (Pt A), 310–317. 10.1016/j.ijbiomac.2018.08.107. 30144543

[ref3] Inoue M.; Uchida Y.; Edagawa M.; Hirata M.; Mitamura J.; Miyamoto D.; Taketani K.; Sekine S.; Kawauchi J.; Kitajima S. The stress response gene is a direct target of the Wnt/β-catenin pathway and inhibits the invasion and migration of HCT116 human colorectal cancer cells. PLoS One 2018, 13 (7), e0194160 10.1371/journal.pone.0194160. 29966001 PMC6028230

[ref4] Li X.; Xiang Y.; Li F.; Yin C.; Li B.; Ke X. WNT/beta-Catenin Signaling Pathway Regulating T Cell-Inflammation in the Tumor Microenvironment. Front Immunol 2019, 10, 2293 10.3389/fimmu.2019.02293. 31616443 PMC6775198

[ref5] Wolfgang C. D.; Chen B. P.; Martindale J. L.; Holbrook N. J.; Hai T. gadd153/Chop10, a potential target gene of the transcriptional repressor ATF3. Mol. Cell. Biol. 1997, 17 (11), 6700–6707. 10.1128/MCB.17.11.6700. 9343434 PMC232524

[ref6] Lu D.; Wolfgang C. D.; Hai T. Activating transcription factor 3, a stress-inducible gene, suppresses Ras-stimulated tumorigenesis. J. Biol. Chem. 2006, 281 (15), 10473–10481. 10.1074/jbc.M509278200. 16469745

[ref7] Jadhav K.; Zhang Y. Activating transcription factor 3 in immune response and metabolic regulation. Liver Res. 2017, 1 (2), 96–102. 10.1016/j.livres.2017.08.001. 29242753 PMC5724780

[ref8] Krylov D.; Barchi J.; Vinson C. Inter-helical interactions in the leucine zipper coiled coil dimer: pH and salt dependence of coupling energy between charged amino acids. J. Mol. Biol. 1998, 279 (4), 959–972. 10.1006/jmbi.1998.1762. 9642074

[ref9] Acharya A.; Rishi V.; Vinson C. Stability of 100 homo and heterotypic coiled-coil a-a ’ pairs for ten amino acids (A, L, I, V, N, K, S, T, E, and R). Biochemistry 2006, 45 (38), 11324–11332. 10.1021/bi060822u. 16981692

[ref10] Acharya A.; Ruvinov S. B.; Gal J.; Moll J. R.; Vinson C. A heterodimerizing leucine zipper coiled coil system for examining the specificity of a position interactions: amino acids I, V, L, N, A, and K. Biochemistry 2002, 41 (48), 14122–14131. 10.1021/bi020486r. 12450375

[ref11] Grigoryan G.; Keating A. E. Structural specificity in coiled-coil interactions. Curr. Opin. Struct. Biol. 2008, 18, 477 10.1016/j.sbi.2008.04.008. 18555680 PMC2567808

[ref12] Fletcher J. M.; Bartlett G. J.; Boyle A. L.; Danon J. J.; Rush L. E.; Lupas A. N.; Woolfson D. N. N@a and N@d: Oligomer and Partner Specification by Asparagine in Coiled-Coil Interfaces. ACS Chem. Biol. 2017, 12 (2), 528–538. 10.1021/acschembio.6b00935. 28026921

[ref13] Zhu B. Y.; Zhou N. E.; Kay C. M.; Hodges R. S. Packing and hydrophobicity effects on protein folding and stability: effects of beta-branched amino acids, valine and isoleucine, on the formation and stability of two-stranded alpha-helical coiled coils/leucine zippers. Protein Sci. 1993, 2 (3), 383–394. 10.1002/pro.5560020310. 8453376 PMC2142373

[ref14] Lau S. Y.; Taneja A. K.; Hodges R. S. Synthesis of a model protein of defined secondary and quaternary structure. Effect of chain length on the stabilization and formation of two-stranded alpha-helical coiled-coils. J. Biol. Chem. 1984, 259 (21), 13253–13261. 10.1016/S0021-9258(18)90686-1. 6490655

[ref15] Crooks R. O.; Lathbridge A.; Panek A. S.; Mason J. M. Computational Prediction and Design for Creating Iteratively Larger Heterospecific Coiled Coil Sets. Biochemistry 2017, 56 (11), 1573–1584. 10.1021/acs.biochem.7b00047. 28267310

[ref16] Wiseman T.; Williston S.; Brandts J. F.; Lin L. N. Rapid measurement of binding constants and heats of binding using a new titration calorimeter. Anal. Biochem. 1989, 179 (1), 131–137. 10.1016/0003-2697(89)90213-3. 2757186

[ref17] Sha H.; Zhang D.; Zhang Y.; Wen Y.; Wang Y. ATF3 promotes migration and M1/M2 polarization of macrophages by activating tenascin-C via Wnt/beta-catenin pathway. Mol. Med. Rep 2017, 16 (3), 3641–3647. 10.3892/mmr.2017.6992. 28714032

[ref18] Khuu C. H.; Barrozo R. M.; Hai T.; Weinstein S. L. Activating transcription factor 3 (ATF3) represses the expression of CCL4 in murine macrophages. Mol. Immunol. 2007, 44 (7), 1598–1605. 10.1016/j.molimm.2006.08.006. 16982098

[ref19] Havranek J. J.; Harbury P. B. Automated design of specificity in molecular recognition. Nat. Struct. Biol. 2003, 10 (1), 45–52. 10.1038/nsb877. 12459719

[ref20] Mason J. M.; Arndt K. M. Coiled coil domains: stability, specificity, and biological implications. Chembiochem 2004, 5 (2), 170–176. 10.1002/cbic.200300781. 14760737

[ref21] Mason J. M.; Schmitz M. A.; Muller K. M.; Arndt K. M. Semirational design of Jun-Fos coiled coils with increased affinity: Universal implications for leucine zipper prediction and design. Proc. Natl. Acad. Sci. U.S.A. 2006, 103 (24), 8989–8994. 10.1073/pnas.0509880103. 16754880 PMC1482553

[ref22] Crooks R. O.; Baxter D.; Panek A. S.; Lubben A. T.; Mason J. M. Deriving Heterospecific Self-Assembling Protein-Protein Interactions Using a Computational Interactome Screen. J. Mol. Biol. 2016, 428 (2 Pt A), 385–398. 10.1016/j.jmb.2015.11.022. 26655848 PMC4751974

[ref23] Yu M.; Ghamsari L.; Rotolo J. A.; Kappel B. J.; Mason J. M. Combined computational and intracellular peptide library screening: towards a potent and selective Fra1 inhibitor. RSC Chem. Biol. 2021, 2 (2), 656–668. 10.1039/D1CB00012H. 34458807 PMC8341738

[ref24] Lathbridge A.; Mason J. M. Computational Competitive and Negative Design To Derive a Specific cJun Antagonist. Biochemistry 2018, 57 (42), 6108–6118. 10.1021/acs.biochem.8b00782. 30256622

