# Supplementary material for: Exponential Combination of a and e/g Intracellular Peptide Libraries Identifies a Selective ATF3 Inhibitor
Source: ACS Chem Biol. 2024 Feb 27;19(3):753–62. doi: 10.1021/acschembio.3c00779 (PMC10949195; doi:10.1021/acschembio.3c00779)
Supplement: Supplementary file 1 — cb3c00779_si_001.pdf [file cb3c00779_si_001.pdf]

# Supporting Information

# Exponential Combination of *a* and *e/g* Intracellular Peptide Libraries Identifies a Selective ATF3 Inhibitor

*Miao Yu[1], T.M. Simon Tang[1], Lila Ghamsari[2], Graham Yuen[2], Claudio Scuoppo[2], Jim A. Rotolo[2], Barry J. Kappel[2], and Jody M. Mason[1*]*

[1]Department of Life Sciences, University of Bath, Claverton Down, Bath, BA2 7AY, UK

[2]Sapience Therapeutics, Inc. 520 White Plains Rd, Tarrytown, NY 10591

[*]Corresponding author: Jody M. Mason (E: j.mason@bath.ac.uk; T: +441225386867)

Keywords: Coiled coil; activating transcription factor/cAMP-dependent transcription factor; ATF3; Transcription Factor; Protein-fragment Complementation; Peptide libraries; Protein-protein interactions

**Fig S1:** Constructed PCA library ***a*** and library ***e/g*** are shown, along with two sequences verified by DNA sequencing to check accuracy and sequence variation

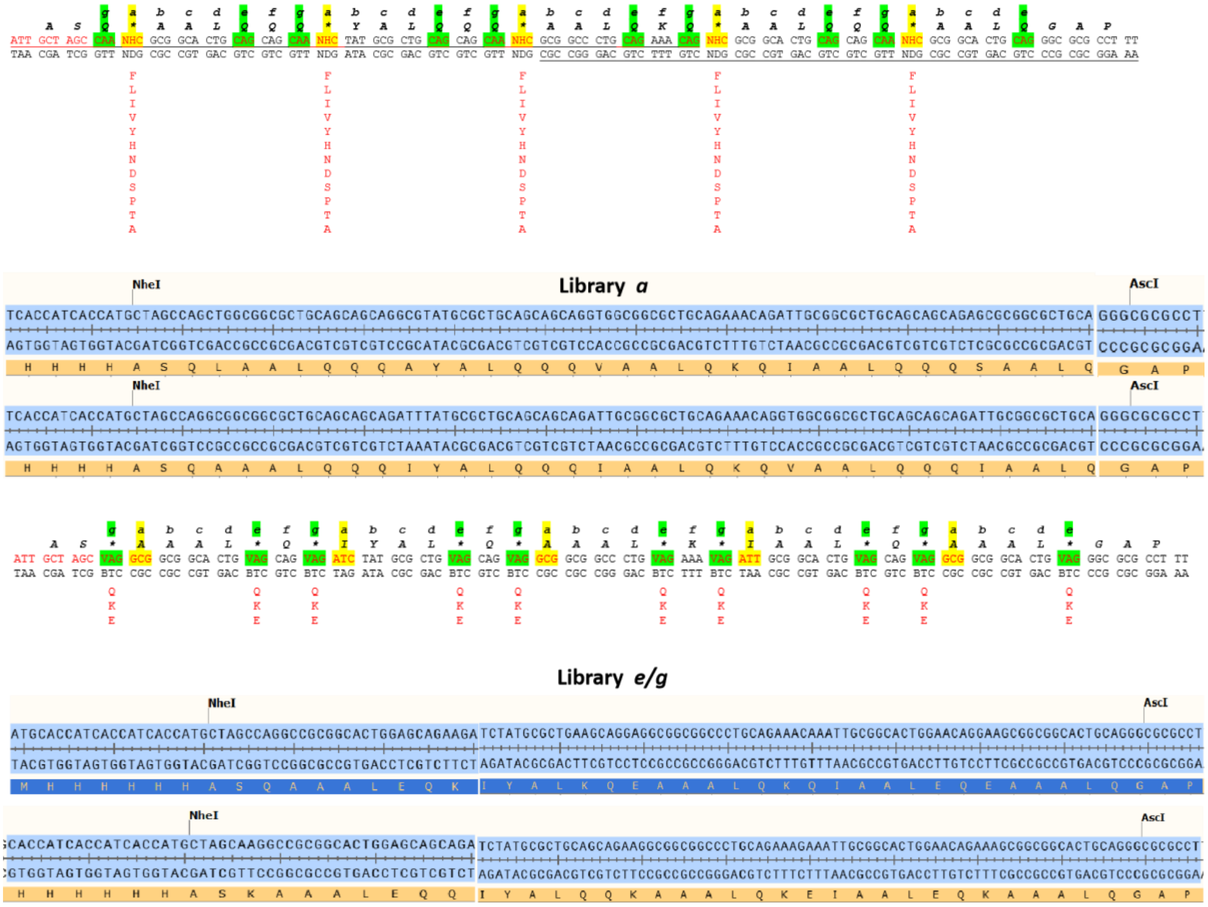

**Fig S2:** DNA sequencing results of competitive PCA library pools for passages 0 to 7. Both single-step selection P0 and competition selections (P1 to P7) are shown in this list. The peptide sequence QLAALQQQAYALQQQNAALQKQVAALQQQIAALQ in the library *a* was seen to dominate at passage 7; and the peptide sequence EAAALEQKIYALKQEAAALEKEIAALEQKAAALK in the library *e/g* was seen to dominate at passage 7.

### The competitive selections of Library *a*

```
P0:    Q L A A L Q Q Q A Y A L Q Q Q N A A L Q K Q V A A L Q Q Q I A A L Q
P0-1:  Q V A A L Q Q Q T Y A L Q Q Q D A A L Q K Q S A A L Q Q Q Y A A L Q
P0-2:  Q A A A L Q Q Q P Y A L Q Q Q Y A A L Q K Q H A A L Q Q Q D A A L Q
P0-3:  Q I A A L Q Q Q L Y A L Q Q Q T A A L Q K Q I A A L Q Q Q A A A L Q
P1:    Q V A A L Q Q Q I Y A L Q Q Q A A A L Q K Q V A A L Q Q Q L A A L Q
P1-1:  Q A A A L Q Q Q Y Y A L Q Q Q I A A L Q K Q A A A L Q Q Q T A A L Q
P1-2:  Q F A A L Q Q Q H Y A L Q Q Q V A A L Q K Q D A A L Q Q Q H A A L Q
P1-3:  Q H A A L Q Q Q D Y A L Q Q Q F A A L Q K Q P A A L Q Q Q N A A L Q
P2:    Q L A A L Q Q Q A Y A L Q Q Q N A A L Q K Q I A A L Q Q Q I A A L Q
P2-1:  Q S A A L Q Q Q P Y A L Q Q Q A A A L Q K Q Y A A L Q Q Q Y A A L Q
P2-2:  Q V A A L Q Q Q V Y A L Q Q Q P A A L Q K Q T A A L Q Q Q F A A L Q
P2-3:  Q P A A L Q Q Q S Y A L Q Q Q V A A L Q K Q N A A L Q Q Q L A A L Q
P3:    Q V A A L Q Q Q V Y A L Q Q Q N A A L Q K Q V A A L Q Q Q I A A L Q
P3-1:  Q A A A L Q Q Q F Y A L Q Q Q S A A L Q K Q I A A L Q Q Q A A A L Q
P3-2:  Q I A A L Q Q Q I Y A L Q Q Q H A A L Q K Q F A A L Q Q Q P A A L Q
P3-3:  Q L A A L Q Q Q A Y A L Q Q Q A A A L Q K Q A A A L Q Q Q I A A L Q
P4:    Q L A A L Q Q Q A Y A L Q Q Q N A A L Q K Q V A A L Q Q Q I A A L Q
P4-1:  Q I A A L Q Q Q I Y A L Q Q Q I A A L Q K Q I A A L Q Q Q A A A L Q
P4-2:  Q V A A L Q Q Q T Y A L Q Q Q A A A L Q K Q A A A L Q Q Q V A A L Q
P4-3:  Q A A A L Q Q Q A Y A L Q Q Q V A A L Q K Q T A A L Q Q Q I A A L Q
P5:    Q L A A L Q Q Q A Y A L Q Q Q N A A L Q K Q V A A L Q Q Q I A A L Q
P5-1:  Q L A A L Q Q Q I Y A L Q Q Q A A A L Q K Q I A A L Q Q Q L A A L Q
P5-2:  Q V A A L Q Q Q A Y A L Q Q Q I A A L Q K Q A A A L Q Q Q I A A L Q
P5-3:  Q I A A L Q Q Q V Y A L Q Q Q V A A L Q K Q V A A L Q Q Q A A A L Q
P6:    Q L A A L Q Q Q A Y A L Q Q Q N A A L Q K Q V A A L Q Q Q I A A L Q
P6-1:  Q A A A L Q Q Q I Y A L Q Q Q N A A L Q K Q V A A L Q Q Q I A A L Q
P6-2:  Q L A A L Q Q Q V Y A L Q Q Q I A A L Q K Q A A A L Q Q Q I A A L Q
P6-3:  Q L A A L Q Q Q A Y A L Q Q Q N A A L Q K Q V A A L Q Q Q L A A L Q
P7:    Q L A A L Q Q Q A Y A L Q Q Q N A A L Q K Q V A A L Q Q Q I A A L Q
P7-1:  Q L A A L Q Q Q A Y A L Q Q Q N A A L Q K Q V A A L Q Q Q I A A L Q
P7-2:  Q L A A L Q Q Q A Y A L Q Q Q N A A L Q K Q V A A L Q Q Q I A A L Q
P7-3:  Q L A A L Q Q Q A Y A L Q Q Q N A A L Q K Q V A A L Q Q Q I A A L Q
```

### The competitive selections of Library *e/g*

```
P0:    E A A A L K Q E I Y A L K Q E A A A L E K K I A A L E Q K A A A L K
P0-1:  E A A A L E Q E I Y A L Q Q E A A A L Q K Q I A A L E Q Q A A A L E
P0-2:  Q A A A L K Q K I Y A L K Q K A A A L E K K I A A L Q Q E A A A L K
P0-3:  K A A A L Q Q Q I Y A L E Q Q A A A L K K E I A A L K Q K A A A L Q
P1:    Q A A A L E Q K I Y A L E Q E A A A L Q K E I A A L K Q Q A A A L K
P1-1:  E A A A L Q Q E I Y A L Q Q K A A A L Q K K I A A L K Q K A A A L Q
P1-2:  K A A A L K Q Q I Y A L E Q Q A A A L E K Q I A A L Q Q Q A A A L K
P1-3:  E A A A L E Q K I Y A L Q Q E A A A L K K E I A A L E Q E A A A L E
P2:    E A A A L K Q K I Y A L K Q K A A A L K K E I A A L E Q K A A A L K
P2-1:  Q A A A L E Q E I Y A L E Q K A A A L E K K I A A L Q Q K A A A L E
P2-2:  E A A A L Q Q K I Y A L Q Q Q A A A L Q K E I A A L K Q E A A A L Q
P2-3:  K A A A L E Q Q I Y A L K Q E A A A L K K Q I A A L E Q Q A A A L K
P3:    E A A A L E Q K I Y A L K Q E A A A L E K E I A A L E Q K A A A L K
P3-1:  E A A A L Q Q Q I Y A L E Q Q A A A L Q K Q I A A L K Q E A A A L K
P3-2:  K A A A L K Q K I Y A L Q Q E A A A L Q K E I A A L K Q K A A A L Q
P3-3:  Q A A A L K Q E I Y A L K Q K A A A L K K E I A A L Q Q E A A A L E
P4:    E A A A L E Q K I Y A L K Q E A A A L E K E I A A L E Q K A A A L K
P4-1:  Q A A A L Q Q E I Y A L K Q K A A A L K K E I A A L Q Q K A A A L K
P4-2:  K A A A L Q Q K I Y A L Q Q E A A A L Q K K I A A L K Q Q A A A L K
P4-3:  E A A A L K Q Q I Y A L E Q Q A A A L K K E I A A L E Q Q A A A L Q
P5:    E A A A L E Q K I Y A L K Q E A A A L E K E I A A L E Q K A A A L K
P5-1:  Q A A A L K Q Q I Y A L Q Q Q A A A L Q K E I A A L Q Q K A A A L K
P5-2:  E A A A L E Q K I Y A L K Q K A A A L K K Q I A A L E Q E A A A L K
P5-3:  K A A A L Q Q E I Y A L E Q E A A A L Q K E I A A L E Q E A A A L E
P6:    E A A A L E Q K I Y A L K Q E A A A L E K E I A A L E Q K A A A L K
P6-1:  E A A A L K Q K I Y A L E Q E A A A L E K E I A A L E Q Q A A A L K
P6-2:  K A A A L E Q E I Y A L K Q K A A A L Q K E I A A L K Q K A A A L K
P6-3:  E A A A L E Q K I Y A L K Q E A A A L E K E I A A L E Q K A A A L K
P7:    E A A A L E Q K I Y A L K Q E A A A L E K E I A A L E Q K A A A L K
P7-1:  E A A A L E Q K I Y A L K Q E A A A L E K E I A A L E Q K A A A L K
P7-2:  E A A A L E Q K I Y A L K Q E A A A L E K E I A A L E Q K A A A L K
P7-3:  E A A A L E Q K I Y A L K Q E A A A L E K E I A A L E Q K A A A L K
```

**Fig S3:** Electrospray MS data for ATF3, ATF3W-a, ATF3W-eg and ATF3W-aeg.

ATF3

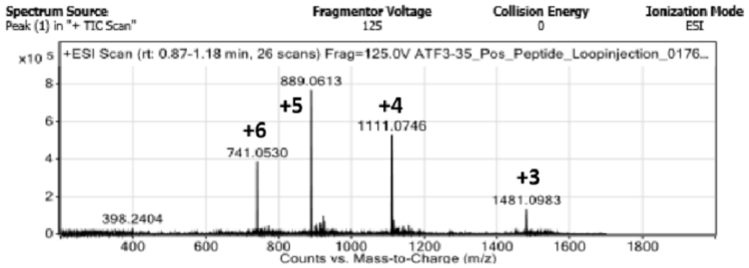


$C_{192}H_{316}N_{52}O_{66}S$

ATF3W-aeg

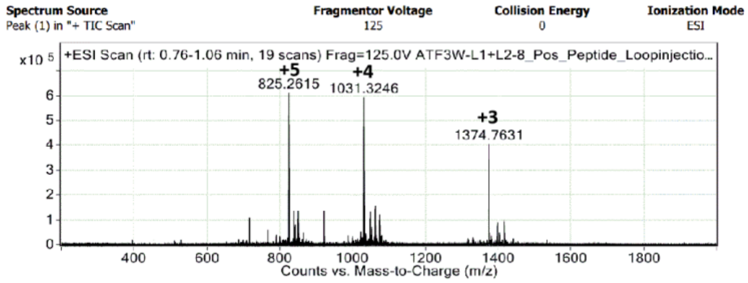


$C_{183}H_{307}N_{49}O_{58}$

ATF3W- a

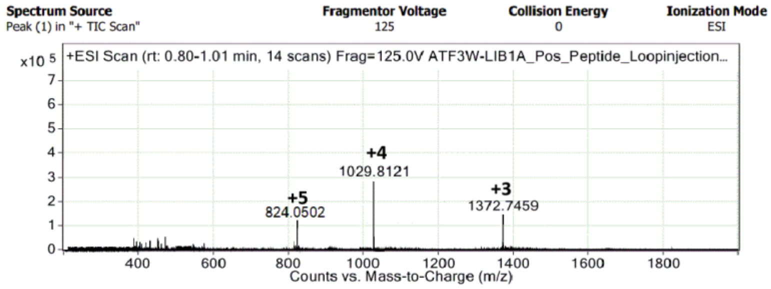


$C_{179}H_{297}N_{55}O_{56}$

ATF3W- eg

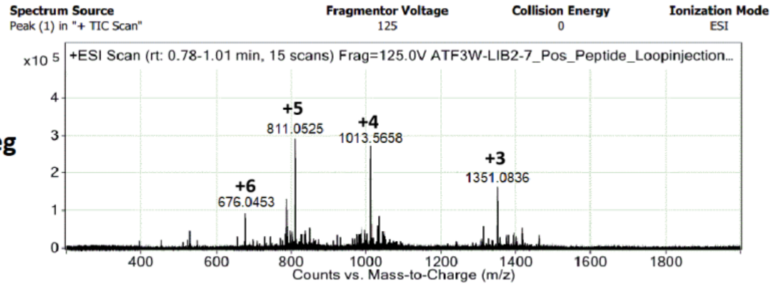


$C_{180}H_{302}N_{48}O_{57}$

**Fig S4:** CD data for ATF3 interacting with ATF3W-a and ATF3W-eg.

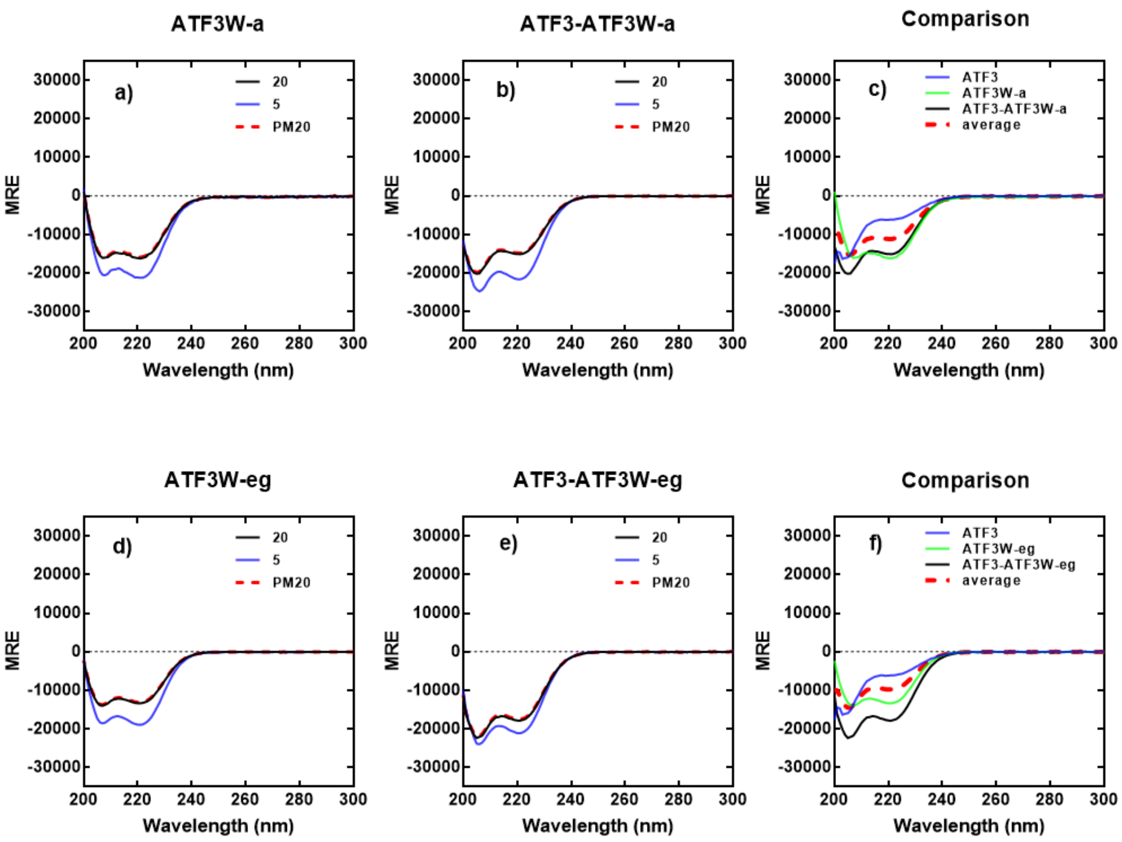


**Peptide permeability assay:** The PAMPA assay measures permeability across an artificial membrane. This method provides an *in vitro* model for passive diffusion. Passive diffusion is an important factor in determining transport through the gastrointestinal tract, penetration of the blood brain barrier, as well as transport across cell membranes. Permeability can also be influenced by several other mechanisms including paracellular transport and active uptake or efflux which are not assessed in PAMPA. Therefore, PAMPA provides a simplistic approach to permeability by only measuring a single mechanism. This avoids the complexities of active transport/efflux and enables the compounds to be ranked on a single permeability property. As the experimental data evidenced in Table S1, ATF3W-aeg peptide exhibited a less efficient permeability of 1.4nm/s and less membrane retention since mass retentions was 5.8% compared to caffeine as control. However, the ATF3W-aeg peptide inhibitor still can be categorised into high permeability as Log(Permeability) of ATF3W-aeg at -5.9 which was larger than -6 according to the industry standard recommended in the manual of the BD™ pre-coated PAMPA plate system.

| Compounds | Permeability(nm/s) | Mass Retention(%) | Log(Pe) |
|---|---|---|---|
| ATF3W-aeg | 1.4 | 5.8 | -5.9 |
| Caffeine | 9.2 | 6.3 | -5.0 |

**TableS1: Shown are peptide permeability assay on ATF3W-aeg. Permeability showed the permeable speed of peptide through membrane and mass retention showed percentage of peptide holding on the membrane.**

**Peptide permeability assay:** To evaluate the peptide permeability, the BD™ pre-coated PAMPA plate system was employed. The 300uL of 200uM peptide in PBS buffer were added in the well of the receiver plate, and slowly placed the filter plate with the 200uL PBS buffer per well on the receiver plate. The peptide concentrations in both plates were determined by UV spectroscopy after the assembly plate system were incubated at room temperature for 5 hours, and the peptide permeability and mass retention were calculated by using the PAMPA plate system formula which recommended in the manufacture manual.

Permeability (in unit of cm/s): $$\frac{-\ln[1 - C_A(t)/C_{equilibrium}]}{A*(1/V_D + 1/V_A)*t}$$

Mass retention: $1 - [\boldsymbol{C}_D(t)*\boldsymbol{V}_D + \boldsymbol{C}_A(t)*\boldsymbol{V}_A]/(\boldsymbol{C}_0 * \boldsymbol{V}_D)$

$\mathbf{C}_0$ = initial compound concentration in donor well (mM)
$\mathbf{C}_D(t)$ = compound concentration in donor well at time t (mM)
$\mathbf{C}_A(t)$ = compound concentration in acceptor well at time t (mM)
$\mathbf{V}_D$ = donor well volume = 0.3 mL
$\mathbf{V}_A$ = acceptor well volume = 0.2 mL
$\mathbf{C}_{equilibrium} = [\boldsymbol{C}_D(t)*\boldsymbol{V}_D + \boldsymbol{C}_A(t)*\boldsymbol{V}_A]/(\boldsymbol{V}_D + \boldsymbol{V}_A)$
**A** = filter area = 0.3 $cm^2$
**t** = incubation time = 18000 s (= 5hr)

**Serum stability:** The quantity of peptide detected by LC-MS is plotted relative to the starting point quantity, demonstrating that the ATF3W-aeg peptide completely degraded over 3-day experimental time course as shown in figure S5.

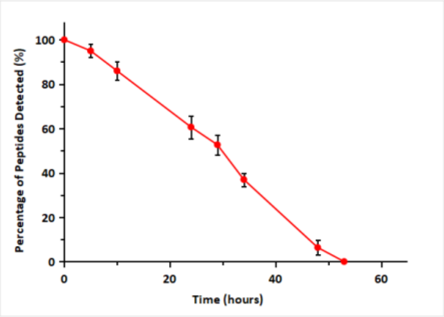


**Fig S5:** Shown are serum stability of ATF3W-aeg. The quantity of peptide detected by LC-MS is plotted relative to the starting point quantity, demonstrating that the ATF3W-aeg peptide completely degraded

over 3-day experimental time course. Data points represent averages of three experimental repeats, with the error bars indicating one SD.

**Serum stability:** Peptide stocks (600 uM) were prepared in water, and 75 uL was added to 1425 uL human serum (Merck) before incubation at 37 °C. 100 uL of aliquots were removed at designated time-points and added to 300 uL of acetonitrile: water (3:1) and centrifuged (18,000 x g, 15 min). The supernatant was analysed by LC-MS, and the peptide was quantified as the sum of the peaks with the two largest intensities[1].

**References:**

(1) Brennan, A.; Leech, J. T.; Kad, N. M.; Mason, J. M. The effect of helix-inducing constraints and downsizing upon a transcription block survival-derived functional cJun antagonist. *Cell Rep Phys Sci* **2022**, *3* (10), 101077. DOI: 10.1016/j.xcrp.2022.101077
